# A Highly Multi‐Stable Meta‐Structure via Anisotropy for Large and Reversible Shape Transformation

**DOI:** 10.1002/advs.202202740

**Published:** 2022-07-21

**Authors:** Giada Risso, Maria Sakovsky, Paolo Ermanni

**Affiliations:** ^1^ Laboratory of Composite Materials and Adaptive Structures Department of Mechanical and Process Engineering ETH Zürich, Leonhardstrasse 21 CH‐8092 Zürich Switzerland; ^2^ Reconfigurable & Active Structures Lab Department of Aeronautics and Astronautics Stanford University Maria Sakovsky CA‐94305 Stanford USA

**Keywords:** mechanical instabilities, programmable, self‐locking, shape adaptation, soft robots

## Abstract

Shape transformation offers the possibility of realizing devices whose 3D shape can be altered to adapt to different environments. Many applications would profit from reversible and actively controllable shape transformation together with a self‐locking capability. Solutions that combine such properties are rare. Here, a novel class of meta‐structures that can tackle this challenge is presented thanks to multi‐stability. Results demonstrate that the multi‐stability of the meta‐structure is strictly tied to the use of highly anisotropic materials. The design rules that enable large‐shape transformation, programmability, and self‐locking are derived, and it is proven that the shapes can be actively controlled and harnessed to realize inchworm‐inspired locomotion by strategically actuating the meta‐structure. This study provides routes toward novel shape adaptive lightweight structures where a metamaterial‐inspired assembly of anisotropic components leads to an unforeseen combination of properties, with potential applications in reconfigurable space structures, building facades, antennas, lenses, and soft robots.

## Graphical abstract

A novel class of meta‐structures that combines large and reversible shape reconfiguration with self‐locking property is presented. This combination of properties is reached by realizing a multi‐stable periodic structure where unit cells can possess up to nine stable states. The multi‐stability is strictly tied to the anisotropy of the materials and can be harnessed to realize a crawling soft robot.

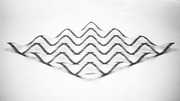

## 1 Introduction

Shape transformation is rapidly appearing at the frontiers of science. In many applications, there is high demand for structural components that are suitable for a wide range of tasks rather than being strictly tied to a unique operational mode. Shape transformation enables structures to drastically alter their 3D shape and, therefore, it enables the design of multi‐functional components that can adapt to different environmental conditions and operate more efficiently. For example, adaptive facades have been investigated for increasing the energy efficiency of buildings,^[^ ^1^, ^2^, ^3^ ^]^ morphing wings for more efficient flight performance,^[^ ^4^, ^5^, ^6^ ^]^ and highly packageable components for better transportation.^[^ ^7^, ^8^ ^]^ Despite significant advancements, for actual applications, most concepts lack needed functionalities. The potential of shape transformation is fully exploited if reversible. To date, most structures can be reconfigured only once,^[^ ^7^, ^9^, ^10^, ^11^, ^12^, ^13^ ^]^ or, if reversible shape change is enabled, they do not have self‐locking capabilities, i.e, continuous power input is necessary to hold desired configurations.^[^ ^14^, ^15^, ^16^ ^]^ Other concepts, that can accommodate large‐shape change, rely on materials that limit the application range.^[^ ^12^, ^17^ ^]^ Successfully integrating reversibility and self‐locking capabilities in large‐shape reconfigurable structures is an open challenge.

Multi‐stability offers the possibility to tackle this challenge. Multi‐stable components intrinsically can switch reversibly between different configurations that correspond to the stable states of the structure, and can self‐lock in any of these configurations.^[^ ^18^ ^]^ To date, only a few concepts enable large shape transformation together with a high number of stable states.^[^ ^19^ ^]^ Assembling bi‐stable elements into periodic structures is a common method to create multi‐stable systems.^[^ ^20^, ^21^ ^]^ However, this can lead to the suppression of stable states,^[^ ^22^ ^]^ or result in complex mechanisms that make the structure difficult to control.^[^ ^23^ ^]^ Other approaches see a significant reduction of the deformations in periodic arrangements compared to those seen in the unit cell alone^[^ ^24^ ^]^ or high coupling between stable states.^[^ ^25^ ^]^

Here, we present a novel class of shape reconfigurable structures that are highly multi‐stable. Our approach synergetically combines three design features well known by the research community: self‐formation, mechanical meta‐materials, and high anisotropy. Each of these features plays a vital role in the design of our concept. Self‐forming structures are 2D components that, when actuated, morph into complex 3D shapes.^[^ ^26^ ^]^ This the modular assembly of conventional materials can lead to properties not achievable with the constituent materials alone.^[^ ^27^, ^28^ ^]^ Global geometry and mechanical properties of the metamaterial can be programmed by tailoring the design parameters of the individual modules. Last but not least, high anisotropy is a property widely used to enhance the functionality and performance of structural components.^[^ ^29^, ^30^ ^]^ This property can be exploited to achieve multi‐stability, as observed in other concepts.^[^ ^18^, ^24^, ^25^ ^]^

**Figure 1:**
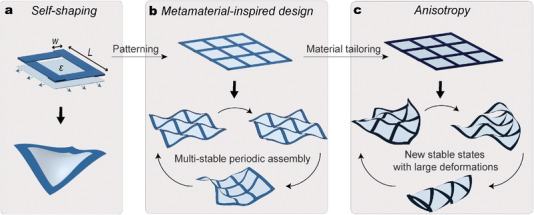
Design of highly reconfigurable and multi‐stable meta‐structures. a) 2D assembly of a unit cell and its lowest energy configuration. b) Periodic combination of the unit cells results in a metamaterial‐inspired design. The meta‐structures show new types of stable states. c) The number of 3D stable shapes is increased by tailoring the frame material. Highly anisotropic composite strips lead to additional stable shapes enabling large deformations.

If these three design features have alone motivated outstanding research advancement, in this work we demonstrate that their combination enables highly versatile shape transformation (**Figure** **1**a–c). The meta‐structure is manufactured by combining 2D periodic assemblies of highly anisotropic strips with pre‐stretched soft membranes. The structure, self‐shapes into a wide range of complex 3D geometries, is programmable, reversibly transformable, and self‐locks into different 3D shapes thanks to its multi‐stability property. Particularly, we prove that the multi‐stability is strictly tied to the anisotropy of the strips. We conclude the work by demonstrating that the 3D shapes can be actively controlled by selectively placing custom‐designed pneumatic actuators on the surface of the meta‐structure. The large‐shape transformation is then harnessed to create inchworm‐inspired locomotion.

## 2 Results

### 2.1 Characterization of a Unit Cell

We construct a unit cell by combining four identical strips with an equi‐biaxially pre‐stretched soft membrane as illustrated in Figure 1a. The two components are bonded together while flat with a double‐sided adhesive tape and, once the pre‐stress of the membrane is released, the cell self‐shapes into a 3D structure characterized by four strips showing the same type of buckled shape (Experimental Section). The out‐of‐plane deformation of the strips can be tailored by varying material and geometrical parameters of the unit cell,^[^ ^31^ ^]^ allowing for a highly versatile design and simple fabrication.

**Figure 2:**
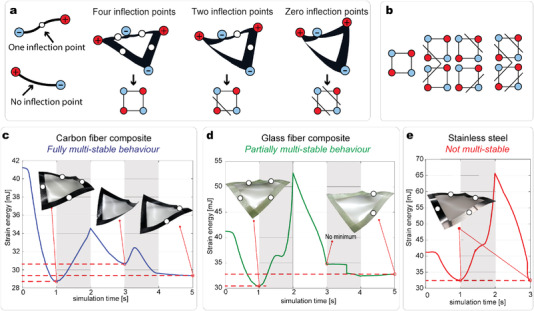
Analysis of the stability of a unit cell. a) Schematic of a unit cell in its main three stable configurations. Each state is characterized by its finite number of inflection points. A schematic illustration of the each state is introduced, where a black diagonal line crosses the two strips that are in the configuration with zero inflection points. b) The seven stable configurations of a fully multi‐stable unit cell. c) Total strain energy profiles of a cell that is manufactured from carbon fiber composite. The cell shows a fully multi‐stable behavior with three energy minima. d) Energy profile of a cell manufactured from glass fiber composite. The cell has a partially multi‐stable behavior with two distinct minima. e) The energy profile of a cell made from stainless steel that possesses only one main stable configuration.

With this method, we can realize multi‐stable unit cells. **Figure** **2**a describes the three main stable states that a unit cell can possess. To characterize the different stable states, we introduce the definition of an inflection point *P*, as a point where the curvature along the strip, κ_1_, satisfies the following condition:

(1) $$\kappa_{1}\left(P\right)=0\wedge \kappa_{1}\left(P-\delta \right)*\kappa_{1}\left(P+\delta \right)<0$$

The stable state that arises when the prestress is released is characterized by a total of four inflection points and consecutive corners lie at different out‐of‐plane levels (blue (‐) and red (+)). If we apply an out‐of‐plane displacement to one of the corners, the two strips connected to this corner will snap to another stable state. This state is characterized by two strips having different types of deformation and no inflection points. In this stable state, the cell possesses a total of two inflection points. The third stable state does not possess any inflection point, resulting in two opposite corners of the cell being snapped. As a consequence of the symmetry of the deformations, a unit cell has fourteen stable configurations in total. Seven of these are schematically represented in Figure 2b. The other seven stable states are symmetric relative to the ones illustrated in Figure 2b, with inverted blue (+) and red (‐) corners (Figure S4, Supporting Information).

We now investigate the influence of the strips' material on the multi‐stability of a unit cell. We compare the behavior of three cells made of three different materials, namely a carbon fiber composite, a glass fiber composite, and stainless steel. To better compare the results, we tailor the total thickness of the strips, *t*, to obtain equivalent longitudinal bending stiffness, *EI*, which is one of the main parameters that drive the out‐of‐plane deformation of the strips.^[^ ^31^ ^]^ This choice of materials leads to a significant variation of the torsional stiffness, *GJ* (Experimental Section).

We characterize the multi‐stability behavior of the three cells with Finite Element (FE) analysis and experiments (Figure 2(c–e)). Stable states are defined as minima of the total strain energy of the structure. To compute the energy landscape of each cell, we utilize the commercial software Abaqus^[^ ^32^ ^]^ and model the different stable states by applying a displacement to one or two opposite corners of the cell. The sequential reconfiguration of the cell is performed in different steps of the simulation, where each step corresponds to 1 s of the fictitious simulation time of the strain energy plot (Experimental Section; Section S1, Supporting Information). The three cells have a very different stability behavior. The carbon fiber unit cell possesses three different energy minima, corresponding to the three stable states of Figure 2c. We define this behavior as fully multi‐stable'. The glass fiber cell possesses two minima (stable states with four and two inflection points), 'partially multi‐stable'. The steel cell is 'not multi‐stable'; it has only one energy minimum, corresponding to the state with four inflection points.

Each of the three cells shows the stable state with four inflection points, which corresponds to the lowest energy level, and is the one arising once the prestress of the membrane is released. The second‐lowest energy level corresponds to the state with two inflection points, which is stable for both the carbon fiber and glass fiber composite cells. The highest energy minimum is one of the stable state with zero inflection points, being stable only with the carbon fiber composite strips. In Video S1 (Supporting Information), we show the different stability behavior of the three cells by manually snapping them. The results validate the FE simulations.

### 2.2 Anisotropy Enhances Multi‐Stability

The previous investigation has shown that a strip in the zero inflection points stable state possesses a high twist (Figure S5, Supporting Information). By utilizing highly anisotropic materials, we can drastically decrease the torsional stiffness of the strips while maintaining the longitudinal bending stiffness unchanged. This results in lower forces needed to impose twisting curvatures. As *GJ* is decreased, the multi‐stability is enhanced, where enhancement indicates that the stable states with two and zero inflection points are enabled. To better understand the design space and draw the requirements for fully multi‐stable behavior, we present a parametric study performed with FE analysis.

**Figure 3:**
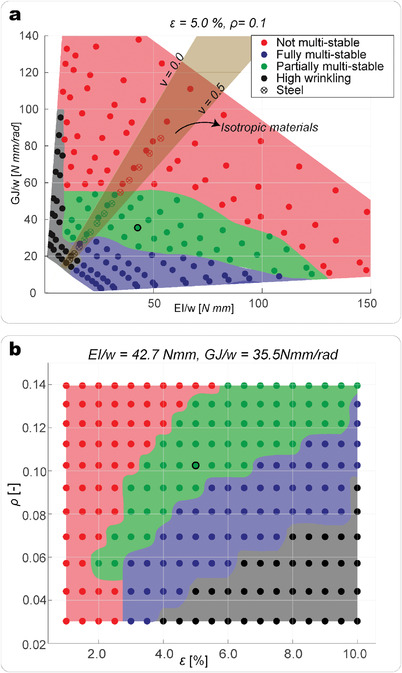
Parametric studies showing the dependency of the multi‐stability behavior on the anisotropy of the strips. a) Multi‐stability design space investigation on unit cells made with carbon fiber composite and stainless steel. Effect of the variation of *EI* and *GJ* for a fixed pre‐stretching and dimensions of the cell are shown. The brown area shows the range of the design space enabled by isotropic materials. b) Design space investigation for constant *EI* and *GJ*. Effects of the variation of the pre‐stretching, ε, and dimensions of the cell are shown.

**Figure** **3**a highlights the dependence of the stability behavior of a unit cell on *EI* and *GJ*, divided by the width of a strip, *w*. To tune the ratio of bending to torsional stiffness, we utilize strips made of carbon fiber composite material and tailor the layup (Experimental Section). Results show that only a small region of the design space enables a fully multi‐stable behavior, as the multi‐stability is highly influenced by the torsional stiffness of the strips. As *GJ*/*w* and *EI*/*w* increase, the multi‐stability is gradually suppressed. For the limit case of strips that are very soft in bending, the out‐of‐plane deformation of the strips increases drastically and wrinkling of the membrane occurs (Figure S6, Supporting Information).

We also investigate the feasibility of realizing fully multi‐stable structures with isotropic materials. The brown area in Figure 3a depicts the area of the design space occupied by isotropic materials, where

(2) $$\frac{GJ/w}{EI/w}=\frac{2}{1+\nu }$$

and ν is the Poisson's ratio, that, for solid isotropic materials is ν ∈ [0.0, 0.5]. Results show that it is possible to realize fully multi‐stable structures with an isotropic material only in a very restricted thickness range.

For the study presented in Figure 3a, the geometry of the strips and pre‐stretching of the membrane have been kept constant. Figure 3b illustrates the multi‐stability behavior of the structure as a function of the aspect ratio of the strips, ρ = *w*/*L*, and the pre‐strain applied to the membrane ε. Increasing the pre‐stretching and/or decreasing the aspect ratio enables a fully multi‐stable behavior. A higher pre‐stretching increases the strain energy stored in the membrane and, therefore, the forces exerted by the membrane on the strips to hold the stable state with zero inflection points. Similarly, a lower aspect ratio implies lower forces for twisting the strips, and, hence, increases the number of stable states.

This result highlights the design rules that ensure a fully multi‐stable behavior of our structure. Composite materials, which can be designed to be highly anisotropic, are ideal candidates to combine multi‐stability with large shape change. Moreover, with composites we can further enhance the multi‐stability behavior by selecting a layup that possesses bend‐twist coupling. In the Section S2 (Supporting Information), we show how bend‐twist coupling enlarges the area of the design space corresponding to fully multi‐stable behavior.

The high anisotropy requirement can also be met by 3D‐printed components and metamaterial design, enlarging the range of applicability of our structures. Recent advances in Additive Manufacturing showed that multi‐material 3D printing allows deposition of materials with different stiffness in the same component.^[^ ^33^ ^]^ Also, novel metamaterials have been demonstrated to possess a very large Poisson ratio,^[^ ^34^ ^]^ which increases the ratio between bending and torsional stiffness (Equation 2).

### 2.3 Periodicity Generates a New Type of Stable State

**Figure 4:**
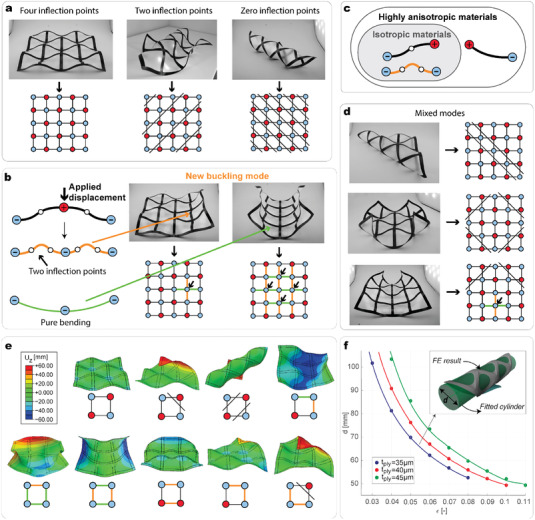
Description of the multi‐stability behavior of a meta‐structure. a) Three stable configurations of a 4 × 4 meta‐structure made with carbon fiber composite material. Each state is characterized by all unit cells having the same type of stable state. b) New type of buckling mode possessed by the meta‐structure. c) Venn diagram illustrating which type of stable states are possessed by a meta‐structure depending on the material utilized (Section S3, Supporting Information). d) Example of some of the many mixed modes of stability of the meta‐structure. e) The nine possible stable states of the internal cell of a 3 × 3 meta‐structure made with carbon fiber composite materials. Geometry and simulation details are given in the Experimental Section. f) Diameter of a 3 × 3 meta‐structure in the stable state with all unit cells having zero inflection points as a function of the pre‐stretching, ε, and ply thickness, *t*_*ply*_.

Having identified the material requirements for a unit cell with a fully multi‐stable behavior, we expand the design by periodicity to realize a meta‐structure. We manufacture a 4 × 4 grid‐like structure made with carbon fiber composite material. In contrast to the prior observations in the literature,^[^ ^22^, ^24^ ^]^ the multi‐stability behavior of the unit cell is preserved in the periodic structure. **Figure** **4**a shows the three stable states of a 4 × 4 meta‐structure characterized by each unit cell having four, two, and zero inflection points, respectively. The stable state with all cells having four inflection points is the one arising once the prestress of the membrane is released. We observe that to have cells with strips with zero inflection points, the deformation of each unit cell must be identical along diagonal lines of the grid (as illustrated in the schematic of Figure 4a). The deformation imposed on the structure to snap from the state with four inflection points to the stable states with zero inflection point is realized by applying an out‐of‐plane force to strips that follow one or multiple diagonals of the grid.

Periodicity, moreover, generates a novel stable state. At a specific intersection point, two aligned strips possess a new buckling mode with two inflection points, while the other two perpendicular strips show an almost pure longitudinal bending, with no inflection points (Figure 4b). The deformation imposed on the structure to snap from the stable state with all cells having four inflection points to this stable state is realized by applying an out‐of‐plane force to one or multiple intersection points of the grid, following horizontal or vertical lines. Comparing our concept to previous ones, such as origami meta‐sheets or composite laminates tessellations,^[^ ^20^, ^35^ ^]^ where periodic arrangements of multi‐stable elements realize multi‐stable structures, only the stable states that a single cell possesses are possible in periodic structure and, in some cases, some of these states are suppressed in periodic structures. The presence of this new type of stable state in our meta‐structure highlights the uniqueness of the approach.

The meta‐structure also possesses mixed modes of stability that include cells with four, two, and zero inflection points simultaneously. Moreover, those states can also co‐exist with the new buckling mode (Figure 4d). Figure 4e illustrates the nine possible stable states of the central unit cell of a 3 × 3 meta‐structure, of which only the first three can be seen in an individual unit cell. To better understand the behavior of a large structure, in Video S2 (Supporting Information), we show how we can manually snap a 6 × 6 meta‐structure following diagonal, vertical, or horizontal lines, and single intersection points.

Interestingly, a meta‐structure manufactured with an isotropic material possesses a stable state with all unit cells having four inflection points and the ones with the new buckling mode (Figure 4c; details in Figure S7, Supporting Information). We can associate the deformation of these strips to the ones of a beam under compression. The periodic assembly of our meta‐structure generates continuity conditions that differ from the homogeneous natural boundary conditions of a single cell, enabling this higher‐order buckling mode to occur. This behavior is independent of the anisotropy of the strips. No twist is observed in those stable states.

To conclude, we illustrate that the global geometry of the meta‐structure can be programmed by tailoring the design parameters. The pre‐stretching, ε, ply‐thickness, *t*_*ply*_, layup, and dimensions can be tailored to fulfill design requirements dictated by different applications. Figure 4f shows how the diameter, *d*, of a 3 × 3 meta‐structure in its stable state with all cells having zero inflection points can be tailored by varying ε and *t*_*ply*_. In a periodic grid, all cells have the same deformation, therefore, the diameter of the cylinder does not depend on the number of unit cells of the meta‐structure. Not accounting for the thickness of the structure itself (≈0.145 mm), an *n* × *n* meta‐structure possesses the same diameter as the 3 × 3. We also observe that in this stable state the composite strips follow a helical pattern. This pattern is similar to those of previous studies where helical‐shaped ribbons are generated via pre‐stretched films or origami folding.^[^ ^36^, ^37^ ^]^ Our approach shows the advantage of being less compliant in compression compared to those concepts, as the composite strips are arranged in a cylindrical lattice geometry. This behavior is comparable to that of composite helical lattices.^[^ ^38^, ^39^, ^40^ ^]^ The combination of a metamaterial‐inspired periodic design and high anisotropy results in highly multi‐stable structures that enable high packaging efficiency and large, reversible, shape transformation.

### 2.4 Actuation

So far, we have shown how to actuate the meta‐structure manually. We now demonstrate that we can control the shape change reversibly with custom‐designed actuators. The actuators need to be light and soft to not affect the multi‐stability and, at the same time, to yield sufficient force output to overcome the energy barrier between the different stable states. To tackle this challenge, we propose a novel pneumatic actuator design. It is inspired by recent research advancements in soft pneumatic actuators for wearable assistive devices and artificial muscles, where high‐power‐to‐weight ratio, stretchability, and large deformations are exploited to assist manifold varieties of sophisticated, large, and repeatable movements.^[^ ^41^, ^42^, ^43^, ^44^, ^45^ ^]^

**Figure 5:**
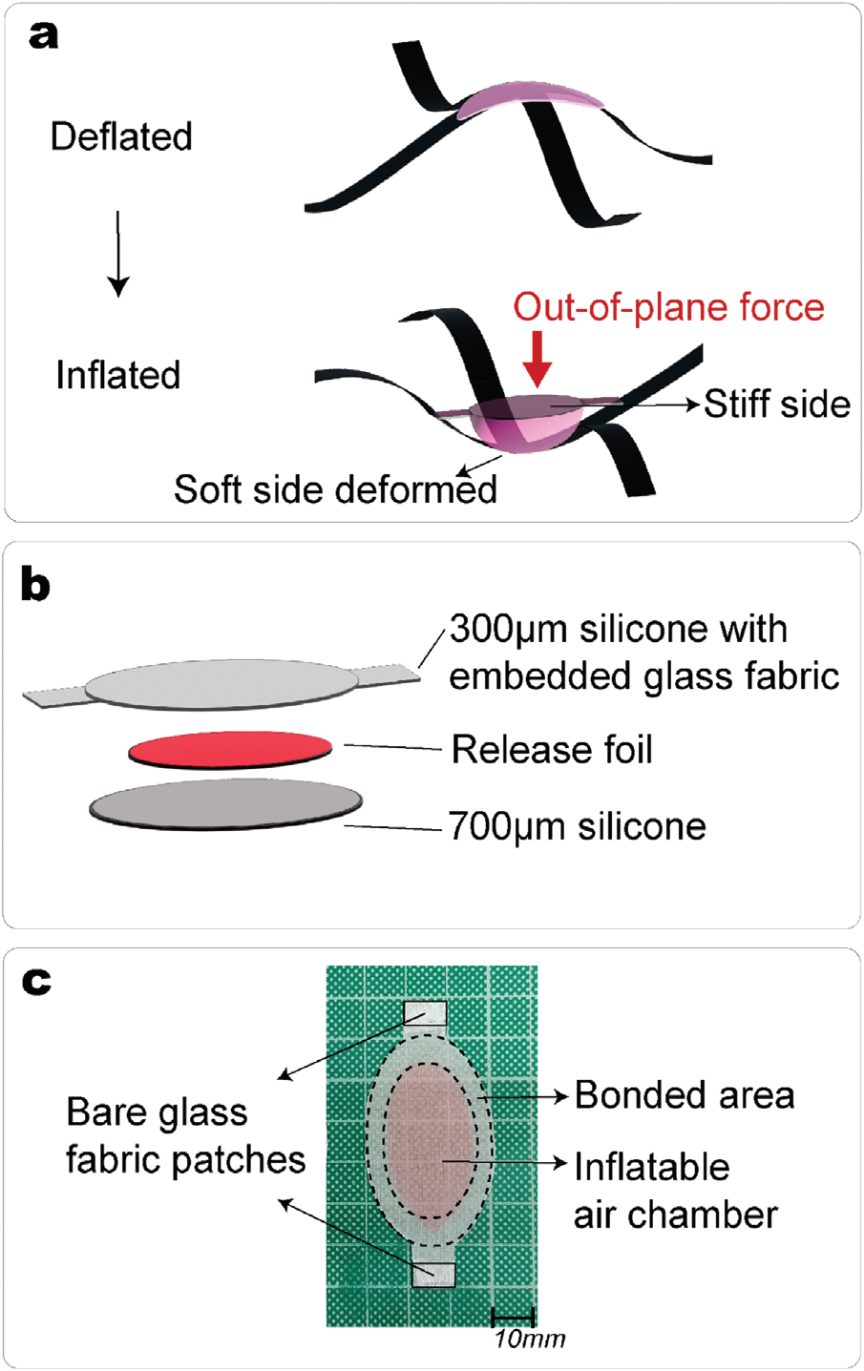
Pneumatic actuator design. a) Schematic of one actuator bonded to the structure. When inflated with air, the actuator deforms asymmetrically, exerting an out‐of‐plane force on the strip that induces snapping to another stable state. b) The actuators are made of three layers, with a total thickness of 1mm. The top layer is significantly stiffer than the bottom one. c) Top view of a manufactured actuator, highlighting the different areas for bonding and inflation.

**Figure 6:**
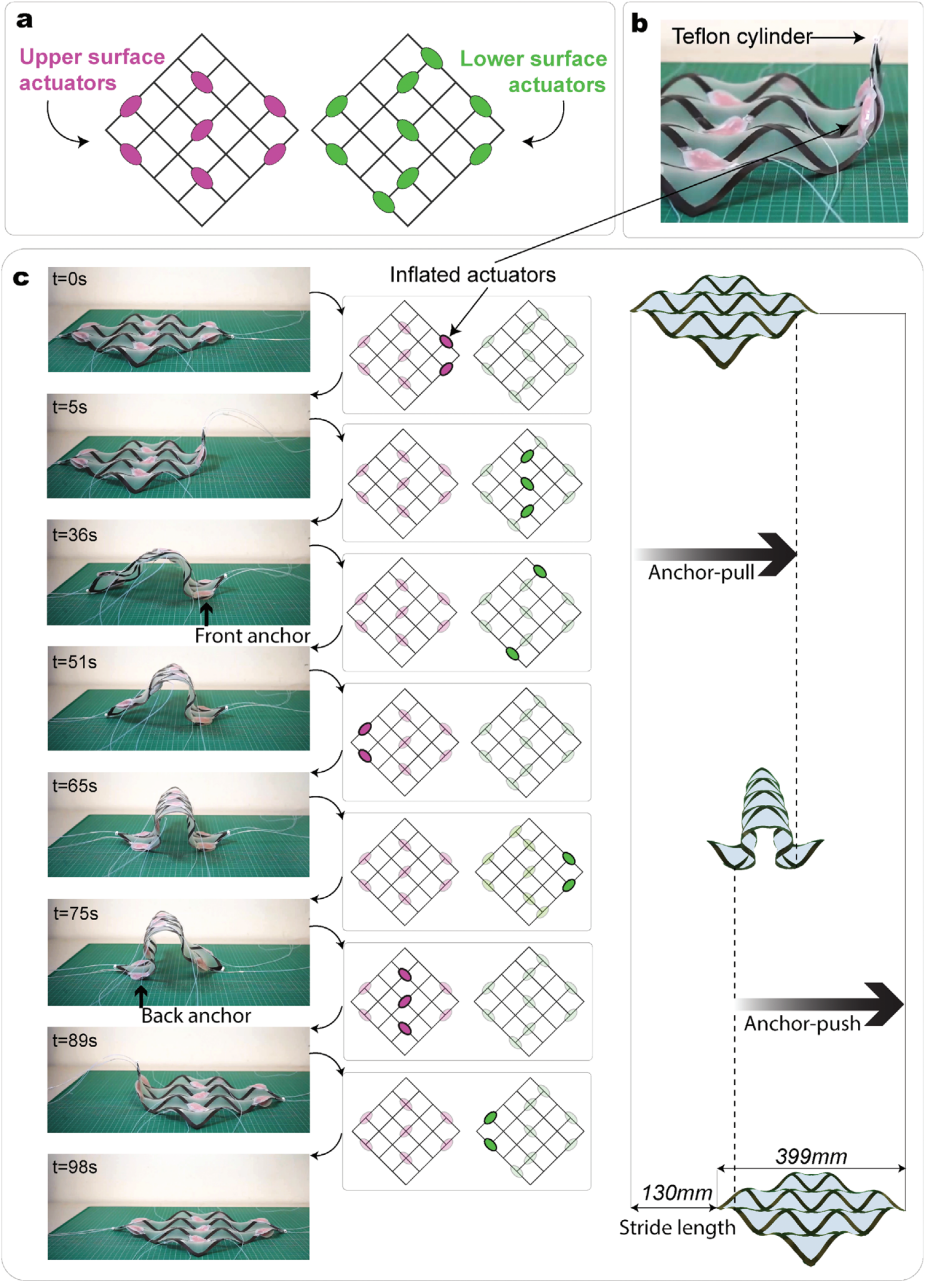
Actuation of the meta‐structure. a) Top and bottom views of the pneumatic actuators placement. b) Detail of two inflated actuators. A small Teflon cylinder is fixed to the structure to enable better sliding. c) Snapshots of the locomotion produced with a 4 × 4 grid with the corresponding inflated actuators for each step. Two full motion cycles are shown in Video S3 (Supporting Information).

We manufacture composite pneumatic actuators composed of two layers and an inflatable air pocket between them (Experimental Section). To induce sufficient forces to generate the snapping of the meta‐structure, we design the actuator to deform asymmetrically. **Figure** **5**a depicts how the asymmetric deformation of an inflated actuator exerts an out‐of‐plane force onto the intersection of a grid. The two layers are manufactured to have a significant difference in elastic modulus ‐ one stiff layer is made of silicone with embedded glass fabric and a soft layer made of silicone only (Figure 5b). Figure 5c shows a top view of an actuator in its deflated state, and **Figure** **6** shows two actuators inflated with a pressure of 0.05 MPa acting on a meta‐structure.

### 2.5 Inchworm‐Inspired Locomotion

The unique characteristics of our meta‐structure make it an ideal candidate for realizing robotic reconfigurable surfaces. New design strategies to realize intelligent soft robots integrating bi‐ or multi‐stable elements have been recently developed.^[^ ^46^ ^]^ Snapping of multi‐stable components can be used to generate propulsion or fast jumping by exploiting the dynamic transition between two stable states.^[^ ^47^, ^48^, ^49^, ^50^ ^]^ Multi‐stability is particularly advantageous because power is required only to induce the snapping of the multi‐stable elements and not throughout the full actuation cycle, and can provide large and repeatable motions with a small actuator strokes. Despite promising advances, it is still difficult to control the transition between stable states^[^ ^46^ ^]^ and ensure multi‐directional maneuverability.^[^ ^51^ ^]^ We exploit the high multi‐stability of our meta‐structure to realize a soft robot with a large stride length and an efficient anchor‐motion mechanism.

We realize inchworm‐like locomotion that shows large stride, and high adaptation to rough and uneven terrains.^[^ ^52^ ^]^ The motion is divided into two parts that are schematized in Figure 6c, right column. In the first part (anchor‐pull), a front anchor is imposed and the backside of the robot is pulled frontward by snapping the two middle diagonals of the grid. In the second part (anchor‐push), a back‐anchor is imposed and the front side of the robot is pushed forward. The crawling mechanism of soft robots is usually enabled by attaching feet‐like devices to the robot to achieve an anisotropic friction effect.^[^ ^53^, ^54^ ^]^ Our concept, instead, takes advantage of the shape transformation of the meta‐structure to achieve the desired front and back anchors. The motion is enabled by snapping the meta‐structure along different diagonal lines, relying on the stable states with zero inflection points, enabled only by highly anisotropic materials.

To realize the motion, we integrate a total of 16 actuators in a 4 × 4 meta‐structure made of carbon fiber composite strips. The actuators are aligned either to vertical or horizontal lines of the grid and bonded to the meta‐structure at the grid intersections in the deflated state. The actuators are placed with the softer side toward the meta‐structure, so when inflated, the deformation of this side forces the meta‐structure to snap. To reversibly change the shape, we position the actuators on both sides of the meta‐structure (Figure 6a). The actuators at the outer left and right sides of the meta‐structure enable alternating holding/sliding of the different parts of the robot (Figure 6b, front/back anchor). Moreover, we place a small Teflon cylindrical tube at the two extremities of the robot to enable better sliding (Figure 6b, top right). Further details on the assembly are given in the Experimental Section.

Figure 6c illustrates the snapshots taken from the full motion of Video S3 (Supporting Information) and the details of the actuation strategy necessary to control the movement. Because of the self‐locking property of the meta‐structure, the actuators do not need to be kept inflated throughout the whole motion, but a vacuum is applied to deflate them as soon as the critical load is reached. The control of the pressure valve, which was carried out manually, highly affects the total locomotion time. The first part of the motion takes a significantly longer time (65 s) because to bend forward, the meta‐structure has to overcome its weight, while the second part, the motion is comparatively quick (33‐s). Despite the dynamics of the snapping between stable states, the locomotion is controlled and repeatable. The locomotion cycle was repeated four times, showing the same stride length. By reversing the sequence of actuation, the robot can move backward. Moreover, placing actuators on the diagonal additionally to those utilized here would allow the robot to move in the direction perpendicular to the current one. Our approach shows a stride length of about a third of its side length (Figure 6c), similar to existing solutions.^[^ ^53^, ^54^ ^]^ Moreover, we can easily expand the design to larger structures, thanks to the periodicity of the meta‐structure.

## 3 Discussion

This paper demonstrates a novel meta‐structure capable of reconfigurable and reversible shape transformation. This rich set of properties is obtained by combining the design features of self‐formation, mechanical metamaterials, and high anisotropy. Our meta‐structure combines a periodic assembly of 2D strips with a soft pre‐stretched membrane to generate mechanical instabilities. The controlled buckling of the strips enables shape transformation that in combination with the multi‐stability property of the meta‐structures, ensures reversibility and self‐locking. By exploring the opportunities that unconventional materials offer, we show that the multi‐stability property is strictly tied to the anisotropy of the strips. Highly anisotropic composite materials enable a new class of stable states that leads to new 3D stable shapes of the meta‐structure. This new class of stable states, characterized by large global deformations, provides the meta‐structure with a very large number of stable states. Moreover, the multi‐stability can be leveraged to design a soft robot that replicates the inchworm locomotion. We demonstrate that the meta‐structure can be actuated by the use of pneumatic actuators and that the transition between stable shapes can be controlled to generate an efficient anchor‐motion mechanism.

The properties of our meta‐material can be harnessed to realize future advances in shape reconfigurable and adaptable devices. We envision potential applications in reconfigurable building facades, antennas and lenses, energy‐absorbing structures, and deployable space structures. Moreover, inspired by recent studies where the multi‐stability is exploited to realize high‐speed actuators,^[^ ^49^, ^50^ ^]^ other types of locomotion that harness the dynamic snapping of the meta‐structure to generate a fast jump could be investigated, realizing robots with multiple locomotion modes. The proposed concept consists of a periodic assembly of square‐like unit cells, but it could be realized with different polygons, to enlarge the range of stable 3D stable shapes. This work opens new fields of research, such as the development of a computational tool that efficiently predicts the stable states of large, non‐periodic structures. Another open possibility is the realization of a novel class of programmable structures where, with new modeling techniques, we can map a multiple of targeted 3D stable shapes to a unique 2D assembly of anisotropic strips.

## 4 Experimental Section

### Fabrication of the Unit Cell

A unit cell consisted of four identical rectangular strips bonded to a bi‐axially pre‐stretched thermoplastic polyurethane (TPU) foil. The strips were connected with each other in a frame‐like square structure with overlapping corners resembling a plain‐weave pattern. Prior to bonding, the corresponding mechanical strains ε_*x*_ = ε_*y*_ = ε were applied to the membrane. The bonding was performed in the flat configuration (*x‐y* plane) and the shape‐forming mechanism arose when the pre‐stress of the foil was released. To hold the membrane in its pre‐stressed state, a large steel frame and some magnets are employed (Figure S8, Supporting Information). The foil used was the Convestro Platilon U2102A Highly Elastic Polyurethane film with a thickness of 0.025 mm.^[^ ^31^ ^]^ The bonding was performed with a 0.13 mm thick 3M High‐Strength Acrylic Adhesive 300LSE double‐sided adhesive tape.

### Unit Cell Characterization: Material Details

The carbon fiber composite cell was manufactured employing M40J/513 epoxy prepreg from NTPT and a [0]_3_ layup. The glass fiber composite cell consisted of E‐glass/513 epoxy prepreg from NTPT and a [0]_12_ layup. The isotropic cell had been manufactured with stainless steel (1.4310). The combination of materials and layup ensured equivalent longitudinal bending stiffness, *EI*, and significant variation of the torsional stiffness, *GJ*. The three cells in Figure 2(c–e) have the same membrane pre‐stretching, ε=4.4%, strips length, *L* = 100 mm, and, width *w* = 13 mm. To compare the three unit cells manufactured using carbon fiber laminate, glass fiber laminate, and stainless steel, the longitudinal bending stiffness *EI*was defined of the strips as:^[^ ^55^ ^]^

(3) $$EI=\frac{w}{d_{11}}$$

and the torsional stiffness, *GJ*, as:

(4) $$GJ=\frac{4w}{d_{66}}$$

where *w* is the width of the strip and *d*_11_ and *d*_66_ are components of the compliance matrix of a laminate expressed as in:^[^ ^56^ ^]^

(5) $$\left[\begin{array}{c} \varepsilon^{0}\\ \kappa \end{array}\right]=\left[\begin{array}{cc} a & b\\ b & d \end{array}\right]\left[\begin{array}{c} N\\ M \end{array}\right]$$

where $\varepsilon^{0}$ and κ are the mid‐plane strains and curvatures and N and M are the force and moment resultants per unit composite width, respectively. **Table** **1** summarizes the relevant material parameters of the three cells of Figure 2(c–e).

**Table 1:** Relevant material properties of the manufactured unit cells

	*t* [mm]	*EI* [N mm]	*GJ*[N mm rad^‐1^]
Carbon fiber composite	0.120	415.58	34.902
Glass fiber composite	0.216	442.86	185.03
Stainless steel	0.130	452.22	654.46

### Parametric Study Details

For the parametric study in Figure 3b, the unit cells are made with M40J/513 laminate and layup [ + θ, −θ, +θ, −θ] with θ ∈ [0, 45], *t*_*ply*_ ∈ [0.028, 0.055] mm, *L* = 78 mm, and *w* = 8 mm. For the parametric study in Figure 3c, a constant layup of [+ 20, −20, +20, −20], *t*_*ply*_ = 0.040 mm, and vary ε∈[1,10]% and ρ ∈ [0.03, 0.014]was assumed. A constant free area of the membrane $A={\left(\bar{L}-2\bar{w}\right)}^{2}$ with $\bar{L}=78$ mm, $\bar{w}=8$ mm was assumed, where the bar notation indicated a constant value.

### Fabrication of the Meta‐Structures

The materials for the membrane and adhesive utilized to assemble the meta‐structures were identical to those of the unit cell. All the meta‐structures showed in this study were manufactured with pre‐stretching ε=6% and with strips made of M40J/513 laminates with a [0/90/0] layup. This layup ensured high anisotropy and reduced the likelihood of matrix failure due to transverse loading. In the 4 × 4 grid of Figure 4 each strip has a total length of 298 mm and width of 10 mm. The meta‐structures were assembled flat, following a plain‐weave pattern. For the 6 × 6 grid of Video S3 (Supporting Information), each strip has a total length of 442 mm and width of 10 mm.

### FE Model

All the FE models for this study had been modeled by performing a geometrically nonlinear static Finite‐Element Analysis (FEA), implemented in the commercial software ABAQUS 6.14‐1.

To investigate the multi‐stability behavior of the unit cells, the analysis was divided into three main steps. In the first one, the shape‐forming phenomenon was modeled, converging to the stable state with four inflection points. In a second step, the cell was snapped to the stable state with zero inflection points, by imposing displacements to two opposite corners of the structure. Last, the two inflection points stable state was induced. In the energy plots in Figure 2(c–e), the gray areas correspond to the steps where deformations are imposed to the structure, while in the white area the structure is not over‐constrained.

To model the behavior of the 3 × 3 meta‐structure of Figure 4e, nine different analyses were performed by imposing different boundary conditions in the first step of the simulation, to induce a different type of buckled mode in the central unit cell. In a second step, the over‐constrained boundary conditions were removed, leading to the nine different stable states. The grid was built from M40J/513 laminates with a [0/90/0] layup, ply thickness *t*_*ply*_ = 0.040 mm, ε=6%, total length of the strips of 226 mm, and width of 10 mm.

The simulation procedures are explained in more detail in the Sections S1 and S3 (Supporting Information).

### Programmability Study

To compute the diameters required for the parametric study of Figure 4f, the stable state of different 3 × 3 meta‐structures was computed by using M40J/513 laminates with a [0/90/0] layup with three different ply thicknesses *t*_*ply*_ = [0.035, 0.040, 0.045] mm and pre‐stretching ε∈[3,11]%. The FE model results were fitted with the best‐Gaussian fitting function of the GOM Correlate 2018 software.

### Fabrication of the Inflatable Actuators and Robot Assembly

The actuators were manufactured from two‐component silicone elastomer (SCS‐RVT‐3428) and a plain weave E‐glass fabric with thickness of 0.09 mm from GIVIDI Fabrics. To fabricate the actuators, a 200 mm × 200 mm glass plate with two spacers of thickness 0.3 mm fixed at two opposite sides of the plate was used. The glass fabric was placed between the spacers and the first layer of silicone was poured on top. With a large spatula,a pressure was applied to remove air bubbles, impregnate the glass fabric, and even the first silicone layer. Small patches of release foil were positioned on the silicone. After 1 h (pot time), the second layer of steel spacers was positioned on top of the previous ones with a thickness 0.7 mm and the second layer of silicone was poured. As before, with the use of a spatula, the thickness of the silicone was evened, and the remaining air bubbles were removed. The actuators were then cured at room temperature for 24 h. Afterward, they were cut to their final shape (Figure 5c). Strong bonding between the actuators and the meta‐structure was ensured by two patches of bare glass fabric as bonding areas. The two areas that were going to serve as bonding patches with a knife and sandpaper to expose the bare glass fabric were manually scraped. To inflate the air chamber, a small PTFE tube with an outer diameter of 0.67 mm and an inner diameter of 0.25 mm was inserted in the air chamber and connected to a pressure valve. The actuators were then bonded to the structure using Cyanolit 201 glue. The release foil prevented the two layers from bonding during the curing, and was left inside the inflatable air chamber. The meta‐structure was manufactured with strips made of M40J/513 laminates with [0/90/0] layup, pre‐stretching ε=6%, and each strip had a total length of 298 mm and width of 10 mm.

## Conflict of Interest

The authors declare no conflict of interest.

## Author Contributions

All the authors contributed to conceive the project. G.R. performed theoretical development, experiments, and numerical simulations. M.S. and P.E provided guidance throughout the research. G.R. wrote the manuscript with inputs from all authors.

## Supporting information

Supporting Information

Supplemental Video 1

Supplemental Video 2 ‐ part 1

Supplemental Video 2 ‐ part 2

Supplemental Video 3

## Data Availability

The data that support the findings of this study are available in the supplementary material of this article.
