# Supplementary material for: A Highly Multi‐Stable Meta‐Structure via Anisotropy for Large and Reversible Shape Transformation
Source: Adv Sci (Weinh). 2022 Jul 21;9(26):2202740. doi: 10.1002/advs.202202740 (PMC9475508; doi:10.1002/advs.202202740)
Supplement: Supplementary file 1 — Supporting Information [file ADVS-9-2202740-s005.pdf]

ADVANCED SCIENCE
Open Access

Supporting Information

for *Adv. Sci.*, DOI 10.1002/advs.202202740

A Highly Multi-Stable Meta-Structure via Anisotropy for Large and Reversible Shape Transformation

Giada Risso, Maria Sakovsky and Paolo Ermanni*

Supplementary information for *A highly multi-stable meta-structure via anisotropy for large and reversible shape transformation*

Giada Risso*, Maria Sakovsky[a], Paolo Ermanni

Laboratory of Composite Materials and Adaptive Structures, Department of Mechanical and Process Engineering, ETH Zürich, Leonhardstrasse 21, CH-8092 Zürich, Switzerland

[a]Current affiliation: Reconfigurable & Active Structures Lab, Department of Aeronautics and Astronautics, Stanford University, Stanford, California 94305

---

1. FE model of the unit cell: details

The elastic parameters of the TPU foil have been determined using the testing machine at the Institute of Mechanical Systems at ETH Zuerich. The material is considered incompressible and its hyperelastic behavior is characterized with the Neo-Hookean material law. The strain-energy function for this material model is given by [1],

$$W = \frac{\mu}{2}(I_1 - 3) \tag{1}$$

where μ is the shear modulus and I_1 the first invariant of the deformation tensor. The derived material constants are summarized in Table 1.

Table 1: Neo-Hookean incompressible material constants of the TPU foil. Details about the material characterization can be found in [2].

$t_m[mm]$	$\mu[MPa]$
0.025	15.486

Table 2 provides the material data utilized for modeling the strips. The strips are modeled using the built-in composite layup tool in ABAQUS and each ply, with thickness t_{ply}, is modeled with its lamina properties. The adhesive layer, utilized for bonding, is included in the strip's layup. The adhesive is modeled as an isotropic material with Young's modulus and Poisson's ratio as in Table 2.

The simulation is divided in three analyses. In the first one, the shape-forming phenomenon is modeled. Here, the pre-stretching of the membrane is imposed by assigning a pre-defined

*Corresponding author

Table 2: Properties of the materials used to manufacture the experimental specimens for this study [3, 4].

	E_{11} (MPa)	E_{22} (MPa)	G_{12} (MPa)	ν_{12} $(-)$	t_{ply} (mm)	ν_f $(-)$
M40J/513	222000.0	7010.0	4661.0	0.314	0.040	0.60
E-glass/513	40564.0	10884.0	4236.9	0.244	0.018	0.56
Stainless Steel 1.4310	190000.0	190000.0	74803.0	0.27	0.130	–
Adhesive	1.0	1.0	0.38	0.3	0.130	–

stress field σ_x, σ_y in the initial step, calculated as:

$$\sigma_x = \sigma_y = \mu\left(\lambda^2 - \frac{1}{\lambda^4}\right) \tag{2}$$

where $\lambda = 1 + \varepsilon$. The frame and the membrane are bonded together using TIE constraints along the common boundaries. The strips are bonded one to the other with TIE constraints. The analysis converges to the the stable state with four inflection points. In the second one, with a restart analysis, specific displacements fields are imposed, to snap to the state with zero inflection points. Lastly, the cell is then snapped to the stable state with two inflection points.

Figure 1 schematizes the different boundary conditions imposed to the unit cell to perform the analysis. In the bottom-left corner of the cell, four nodes are constrained in all degrees of freedom during the whole simulation time. To move from the state with four inflection points to the one with zero, an out-of-plane displacement is applied to two opposite corners of the structure, while other nodes are hold at their z-displacement (see Figure 1, top-left). Afterwards, the imposed displacements are released and the model converges to the new stable state. To move to the state with two inflection points, we then applied a negative displacement to one to the corners, while fixing the z-displacement of the opposite one (see Figure 1, down-left). An automatic stabilization with constant damping factor 1×10^{-8} command was used from the second step of the analysis to avoid numerical instabilities due to snap-through of the strips. This value has been selected by performing a sensitivity analysis, similarly to other studies [5, 6], and the minimum value required for convergence was selected.

2. Bend-twist coupling effect on the multi-stability of a unit cell

Figure 2 shows the dependence of the stability behavior of a unit cell with $\varepsilon = 5\%$, $L = 78mm$, $w = 8mm$ at the variation of EI and GJ for a layup that shows bend-twist coupling. The analysis is performed on a unit cell with strips with a layup of $[+\theta, -\theta]_s$ of M40J/513 laminate with $\theta \in [0, 45]$, and $t_{ply} \in [0.028, 0.055]\,mm$. This layup shows bend-twist coupling, in fact, the d_{16} component of the compliance matrix is nonzero. Compared to the results with the $[+\theta, -\theta]_s$, a larger area of the design space possesses a fully multi-stable behaviour, demonstrating that laminates with bend-twist coupling term are enhancing the multi-stability.

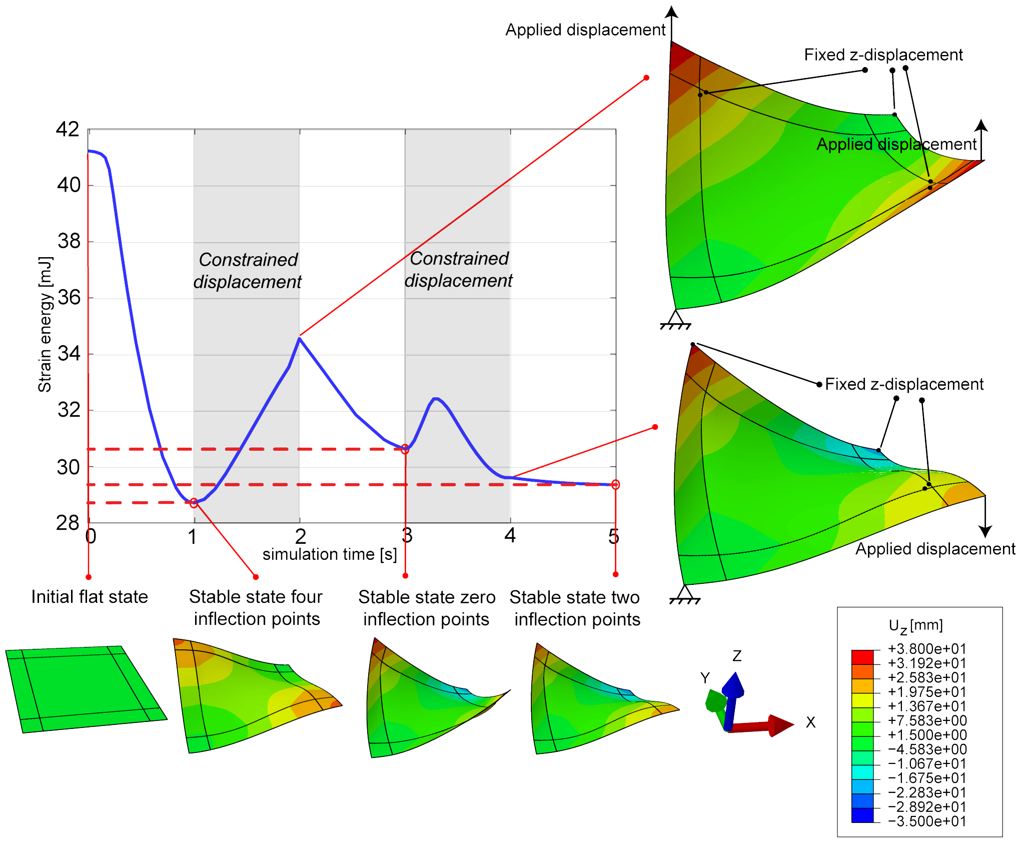

Figure 1: Total strain energy profiles of a cell is manufactured with M40J/513 epoxy pre-preg with $[0]_3$ layup with details about the boundary conditions of the FE model. The cell shows a fully multi-stable behavior with three energy minima.

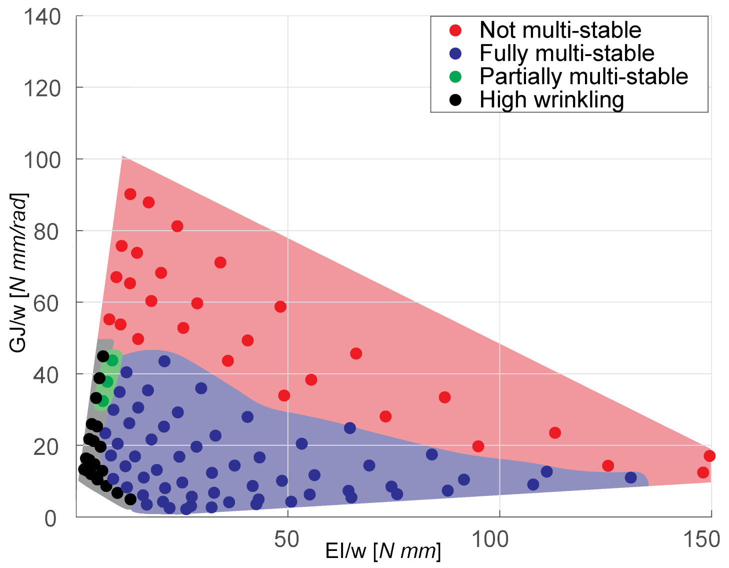

Figure 2: Parametric studies showing the dependency of the multi-stability behavior of a unit cell made with M40J/513 epoxy pre-preg and layup $[+\theta, -\theta]_s$ with $\theta \in [0, 45]$ and $t_{ply} \in [0.028, 0.055]mm$.

3. FE model of the meta-structure: details

The FE results of the nine different stable states of the meta-structure illustrated in Figure 4e of the manuscript are reported here in Figure 3a labeled from **a** to **i**. We implement in the commercial software ABAQUS© 6.14-1 a geometrically nonlinear static Finite-Element Analysis (FEA). Reduced integration, S4R, shell elements are employed for the FRP laminates and M3D4R membrane elements for the TPU foil. For this analysis, the materials have been modeled identically to those of the unit cell. The strips have a layup of [0/90/0] of M40J/513 laminate,ply thickness $t_{ply} = 0.040mm$, total length of the strips 226mm, and width 10mm. The membrane pre-stretching is $\varepsilon = 6\%$.

Nine different analysis are performed by imposing different boundary conditions in the first step of the simulation, to induce a different type of buckled mode in the central unit cell. Table 3 and Table 4 summarize the different boundary conditions imposed in the first step of the analysis for each of the nine analyses (a-i). We call U_x, U_y, U_z the displacements along the x- y- z- axis, respectively, and ϕ_x, ϕ_y, ϕ_z the rotations along the x- y- z- axis, respectively. The notation $h_i v_j$ with $i, j \in [1, 4]$ indicates that the boundary conditions are imposed in the central node of the intersection between the horizontal strip h_i and the vertical strip v_j has illustrated in Figure 3b. In the Table 3 and Table 4, we also illustrate when a an external pressure, p, perpendicular to the x-y plane is applied at the area of intersection of two strips h_i and v_j. In the second step of all the nine analyses, all the boundary conditions are suppressed and for the node $h2v2$ it is enforced that $(u, v, w, \phi_x, \phi_y, \phi_z)$ are fixed at their current value. An automatic stabilization with constant damping factor 1×10^{-8} command was used in the first step of the analysis to avoid numerical instabilities as for the unit cell.

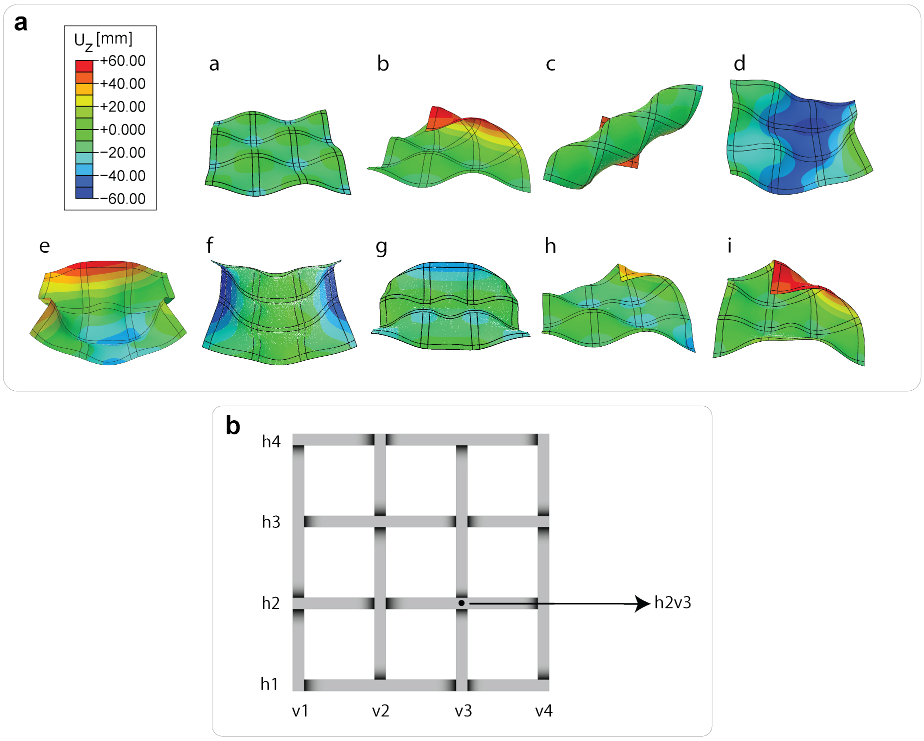

Figure 3: 3 grid analysis. **a**) Nine stable states modeled with FE. **b**) Nomenclature utilized to illustrated the nodes where the boundary conditions are imposed.

Table 3: Summary of the boundary conditions in the first step of the FE analysis for the stable configurations from **a** to **e** of Figure 3

	**a**	**b**	**c**	**d**	**e**
**h1v1**	$(U_x, U_y, U_z) = (0, 0, 0)$	-	$(U_x, U_y, U_z, \phi_x, \phi_y, \phi_z) = (0, 0, 0, 0, 0, 0)$	-	-
**h1v2**	-	$U_z = 0$	-	$p = 0.00001$	-
**h1v3**	$U_z = 0$	-	-	$p = 0.00001$	-
**h1v4**	-	$U_z = 0$	-	-	-
**h2v1**	-	-	-	-	$U_z = 0$
**h2v2**	$U_z = 0$	$(U_x, U_y, \phi_x, \phi_y, \phi_z) = (0, 0, 0, 0, 0)$, $p = 0.001$	$U_z = 0$	$(U_x, U_y, U_z, \phi_z) = (0, 0, 0, 0)$	$(U_x, U_y, U_z, \phi_z) = (0, 0, 0, 0)$
**h2v3**	-	$U_z = 0$	-	$U_z = 0$	$U_z = 0$
**h2v4**	$U_z = 0$	-	-	-	$U_z = 0$
**h3v1**	$U_z = 0$	-	-	-	-
**h3v2**	-	$U_z = 0$	-	$p = 0.001$	-
**h3v3**	$U_z = 0$	-	-	$p = 0.001$	-
**h3v4**	-	-	-	-	-
**h4v1**	-	$U_z = 0$	-	-	$U_z = 0$
**h4v2**	$U_z = 0$	-	-	$U_z = 0$	$U_z = 0$
**h4v3**	-	-	-	$U_z = 0$	$U_z = 0$
**h4v4**	$U_z = 0$	$p = 0.00001$	$U_z = 0$	-	-

Table 4: Summary of the boundary conditions in the first step of the FE analysis for the stable configurations from **f** to **i** of Figure 3

	**f**	**g**	**h**	**i**
**h1v1**	-	$U_z = 0$	$(U_x, U_y, U_z, \phi_x, \phi_y) = (0, 0, 0, 0, 0)$	-
**h1v2**	-	-	-	-
**h1v3**	-	-	-	-
**h1v4**	-	$U_z = 0$	$U_z = 0$	-
**h2v1**	$U_z = 0$	-	-	-
**h2v2**	$(U_x, U_y, U_z, \phi_z) = (0, 0, 0, 0)$	$(U_x, U_y, U_z, \phi_z) = (0, 0, 0, 0)$	$(U_x, U_y, U_z, \phi_x, \phi_y) = (0, 0, 0, 0, 0)$	$(U_x, U_y, U_z, \phi_x, \phi_y) = (0, 0, 0, 0, 0)$
**h2v3**	$U_z = 0$	$(U_y, U_z, \phi_z) = (0, 0, 0)$	$(U_z, \phi_x, \phi_y) = (0, 0, 0)$	$(U_z, \phi_x, \phi_y) = (0, 0, 0)$
**h2v4**	$U_z = 0$	-	-	-
**h3v1**	$U_z = 0$	$U_z = 0$	-	-
**h3v2**	$U_z = 0$	-	$(U_z, \phi_x, \phi_y) = (0, 0, 0)$	$(U_z, \phi_x, \phi_y) = (0, 0, 0)$
**h3v3**	$U_z = 0$	-	-	-
**h3v4**	$U_z = 0$	$U_z = 0$	$U_z = 0$	-
**h4v1**	-	-	$U_z = 0$	-
**h4v2**	-	$U_z = 0$	-	-
**h4v3**	-	$U_z = 0$	$U_z = 0$	-
**h4v4**	-	-	-	-

4. Other figures

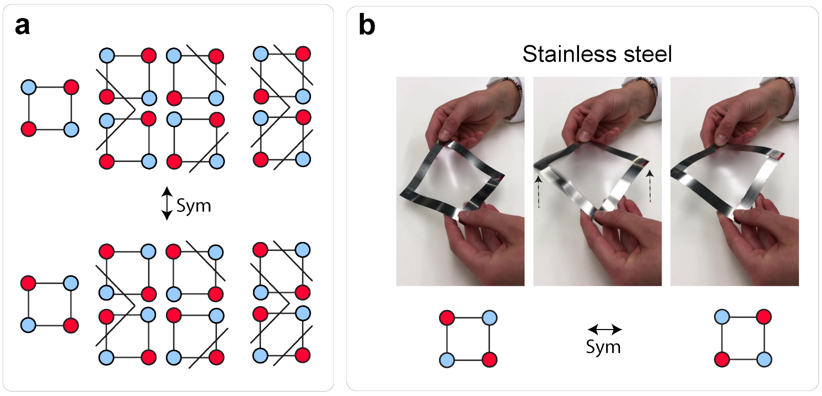

Figure 4: **a)** The 14 stable states of a fully multi-stability unit cell, taking into account the ones that are symmetric one to another. **b)** Snapping between the two stable states of a not multi-stable unit cell made with stainless steel.

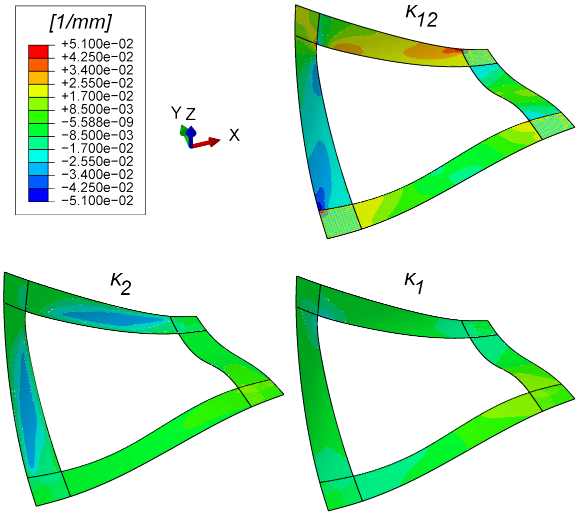

Figure 5: FE model results of a unit cell made of $[0]_3$ layup of M40J/513 laminate in its stable state with total of two inflection points. Plot of the main curvature κ_1, κ_2, and κ_{12} of the strips.

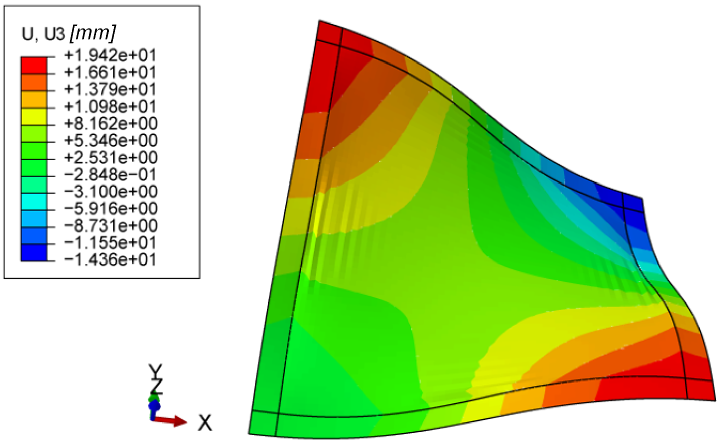

Figure 6: Example of high wrinkling in the membrane in the FE model of a unit cell made with M40J/513 laminate with [+20, −20, +20, −20] layup, ε = 7% and aspect ratio ρ = 0.06 in its stable state with four inflection points.

References

[1] R. S. Rivlin, G. I. Taylor, Large elastic deformations of isotropic materials. ii. some uniqueness theorems for pure, homogeneous deformation, Philosophical Transactions of the Royal Society of London. Series A, Mathematical and Physical Sciences 240 (822) (1948) 491–508.

[2] G. Risso, M. Sakovsky, P. Ermanni, Instability-driven shape forming of fiber reinforced polymer frames, Composite Structures 268 (April) (2020) 113946. doi:10.1016/j.compstruct.2021.113946.

[3] North Thin Ply Technologies, NTPT Thinpreg 513 Data Sheet (2017).

[4] Toray Carbon Fibers America, M40J Data Sheet (2017).

[5] S. Daynes, P. Weaver, Analysis of unsymmetric CFRP-metal hybrid laminates for use in adaptive structures, Composites Part A: Applied Science and Manufacturing 41 (11) (2010) 1712–1718. doi:10.1016/j.compositesa.2010.08.009.

[6] F. Dai, H. Li, S. Du, A multi-stable lattice structure and its snap-through behavior among multiple states, Composite Structures 97 (2013) 56–63. doi:10.1016/j.compstruct.2012.10.016.

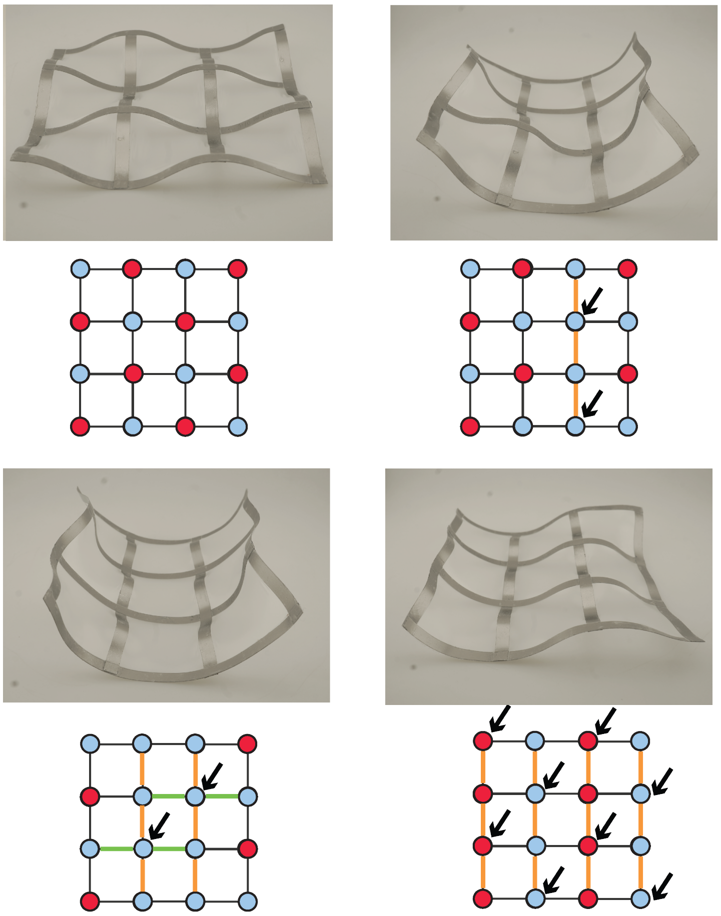

Figure 7: Example of stable states of a 3×3 grid made with Stainless steel strips with total thickness $0.1mm$, pre-stretching $\varepsilon = 5\%$, length $L = 82mm$ and width $w = 10mm$.

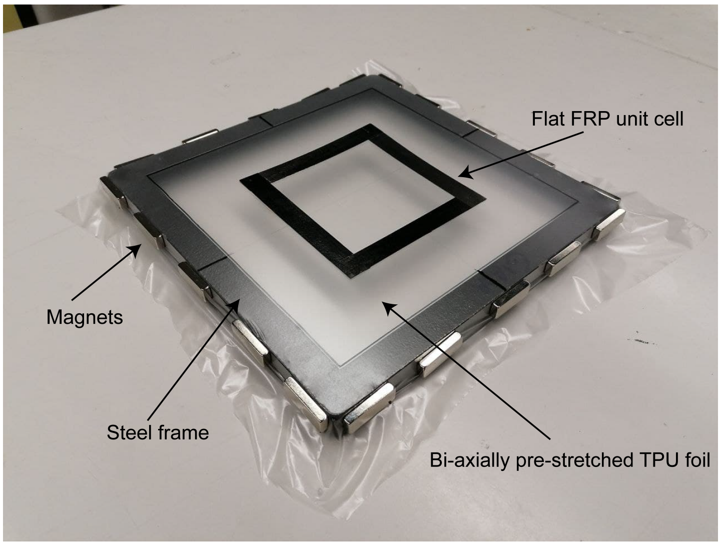

Figure 8: Manufacturing of a unit cell. Bonding of a unit cell to the bi-axially pre-stretched TPU foil prior to the self-shaping of the structure. The TPU foil is stretched manually and hold in its pre-stressed state with the use of a steel frame and some magnets.
